# 1268. A retrospective observational study to evaluate the efficacy of antimicrobial time-out for optimizing the duration of antimicrobial administration in emergency abdominal surgery patients

**DOI:** 10.1093/ofid/ofad500.1108

**Published:** 2023-11-27

**Authors:** Takahiro Suenaga, Hiroaki Hata

**Affiliations:** Kyoto Medical Center, Kyoto, Kyoto, Japan; Kyoto Medical Center, Kyoto, Kyoto, Japan

## Abstract

**Background:**

Appropriate use of antibiotics is important for the prevention of antimicrobial-resistant organisms. However, prolonged administration of broad-spectrum antimicrobial agents is frequently used in surgery, especially in emergency surgery with infection. The low rate of the appropriate use of antimicrobial agents is regarded as a worldwide problem. In order to promote the proper use of antimicrobials, our department has administered daily antimicrobial time-outs to surgical patients as part of the Antimicrobial Stewardship Program since January 2018. This study investigates whether the Antimicrobial Time-out has led to appropriate use of antibiotics.

**Methods:**

Patients aged 18 years and older who underwent emergency abdominal surgery in our department from May 2017 to August 2018 were included. Pre- and postoperative clinical information was extracted from the medical records.

**Results:**

56 and 66 patients were treated before and after the introduction of antimicrobial time-out, respectively. The mean duration of antimicrobial use was 3.8 and 3.3 days before and after the introduction of antibiotic time-out. No significant difference was observed, but a 13.7% decrease was observed (P=0.51). Limiting the analysis to patients who received a minimum of two days of antimicrobial therapy, treated patients were 23 and 27, and the mean duration of antimicrobial use was 7.8 and 6.6 days(P=0.38) before and after the introduction of antibiotic time-out, respectively.

There were also no significant differences in the incidence of complications (Clavien-Dindo classification Grade II or higher) (28.6% vs. 25.8%, P=0.73) or length of hospital stay (17.2 vs. 14.0 days, P=0.38).

**Conclusion:**

Antibiotic time-out in surgical practice may contribute to a reduction in antimicrobial use without increasing complication rates or length of hospital stay.

**Disclosures:**

**All Authors**: No reported disclosures

